# Circulating Tumor Cell-Free DNA as Prognostic Biomarker in Non-Small Cell Lung Cancer Patients Undergoing Immunotherapy: The CORELAB Experience

**DOI:** 10.3390/ijms26020611

**Published:** 2025-01-13

**Authors:** Stefania Gelmini, Adele Calabri, Irene Mancini, Camilla Eva Comin, Valeria Pasini, Marco Banini, Vieri Scotti, Pamela Pinzani

**Affiliations:** 1 Department of Experimental and Clinical Biomedical Sciences “Mario Serio”, University of Florence, 50134 Florence, Italy; stefania.gelmini@unifi.it (S.G.); marco.banini@unifi.it (M.B.); 2 Molecular and Clinical Biochemistry Laboratory, Azienda Ospedaliero-Universitaria Careggi, 50134 Florence, Italy; 3 Department of Experimental and Clinical Medicine, University of Florence, 50134 Florence, Italyvale.pasini@gmail.com (V.P.); 4 Radioterapy Unit, Azienda Ospedaliero-Universitaria Careggi, 50134 Florence, Italy; vieri.scotti@unifi.it

**Keywords:** liquid biopsy, NGS, NSCLC, immunotherapy, prognostic biomarkers, PD-L1

## Abstract

The expression level of Programmed Death-Ligand 1 (PD-L1) determined by the immunohistochemical method is currently approved to test the potential efficacy of immune-checkpoint inhibitors and to candidate patients with Non-Small Cell Lung Cancer (NSCLC) for treatment with immunotherapeutic drugs. As part of the CORELAB (New prediCtivebiOmaRkers of activity and Efficacy of immune checkpoint inhibitors in advanced non-small cell Lung cArcinoma) project, aimed at identifying new predictive and prognostic biomarkers in NSCLC patients receiving immunotherapeutic drugs, we investigated the role of circulating tumor DNA (ctDNA) molecular characterization as an additional predictive biomarker. We analyzed plasma ctDNA by targeted Next Generation Sequencing in a subset of 50 patients at different time points. ctDNA content was inversely correlated with the clinical outcome both at a baseline and after 2 months of treatment. OS was significantly higher in patients with ≥50% ctDNA reduction. *TP53* and *KRAS* were the most frequently mutated genes, and patients with *KRAS* and/or *TP53* mutations showed worse outcomes than patients without detectable variants or with mutations in other genes. Fewer common variants were found in *BRAF*, *EGFR*, *MAP2K1*, *MET*, *NRAS*, and *PIK3CA* genes. Our data demonstrated that molecular characterization of ctDNA and also its quantitative evaluation could serve as a dynamic, real-time prognostic, and predictive biomarker, enabling regular molecular monitoring of therapy efficacy in support of other medical examinations.

## 1. Introduction

Non-Small Cell Lung Cancer (NSCLC) is one of the most common types of malignancies worldwide and the principal cause of cancer-related deaths [1]. The prognosis of NSCLC is challenging due to the unavailability of tools for early-stage diagnosis and the late onset of symptoms in the development of the disease, which limits treatment choices and generally results in poor survival [1].

Treatment for NSCLC is based on surgical resection, chemotherapy, radiotherapy, and targeted therapy, with unsatisfying therapeutic efficacy; more recently, immune checkpoint inhibitors (ICI) have also been used [2].

In fact, evasion of the immune system is one of the many characteristics required for cancer growth, and subverting immune checkpoints is one of the ways in which this can occur, thus providing an opportunity for therapeutic intervention. The two most clinically relevant checkpoints, Cytotoxic T-Lymphocyte Antigen 4 (CTLA-4) and Programmed Cell Death Protein (PD1), act as brakes on the anticancer immune response [3].

Immune checkpoint inhibitors anti-PD1/PD-L1 antibodies, alone or in combination with anti-CTLA-4 antibodies, have shown an increasing implementation in the management of metastatic cancer, such as metastatic melanoma, by increasing both progression-free survival (PFS) and overall survival (OS) [3]. A large number of experimental studies have shown that immune checkpoint inhibitors are safer and more effective than traditional therapeutics and have allowed for the development of better guidance in the clinical treatment of advanced NSCLC patients [2,4]. Either after immunotherapy as monotherapy (first and second line, in conditions with ≥50% PD-L1 expression) or immunotherapy combined with chemotherapy (first line, in patients with <50% PD-L1 expression), the 5-year OS rate was 20% in unselected patients and up to 40–50% in patients with high PD-L1 expression [5].

Unfortunately, many patients do not benefit from immunotherapy. Therefore, the identification of predictive biomarkers for treatment with ICIs in NSCLC patients will not only help to choose patients who will benefit from immunotherapy but also limit non-effective treatments.

Immunohistochemistry is currently assessed and approved as the reference method to evaluate PD-L1 expression in tissue as a predictive factor for the activity and efficacy of immune checkpoint inhibitors in a number of solid tumors, including NSCLC. The use of tumor PD-L1 expression as a biomarker has been studied extensively [6]. In general, across all tumor types, anti-PD-1/PD-L1 therapy results in response rates ranging from 0% to 17% in patients with PD-L1-negative tumors, whereas in those with tumors that express PD-L1, response rates range from 36% to 100% [6]. However, widespread application and standardization of PD-L1 as a biomarker has been limited by the different detection methods used in practice (immunohistochemistry (IHC)), flow cytometry, versus mRNA expression) [7]. In addition, there is no consensus on the cut-off level of PD-L1 expression for positivity. Furthermore, many tumors not only express PD-L1 on malignant cells but also on the nonmalignant cells within the tumor microenvironment. Finally, PD-L1 expression is only applicable to patients treated with PD-1/PD-L1 blockade and not to other types of immunotherapy.

Recently, new biomarkers, including Tumor Mutational Burden (TMB) and plasma-deriving circulating tumor DNA (ctDNA), have been proposed as predictive factors of response and efficacy to immunotherapy in NSCLC and other solid tumors [5,8].

ctDNA is part of a broader framework of Liquid Biopsy (LB), which includes other tumor-derived circulating biomarkers such as circulating cell-free RNA (noncoding, miRNA, and messenger RNA), extracellular vesicles (exosomes and oncosomes), tumor-educated platelets, circulatory proteins; circulating immune cells and immune system components and the circulating microbiome in the blood. The LB concept also covers other physiological fluids such as cerebrospinal fluid, urine, bone marrow, sputum, and saliva [9]. A variety of ctDNA-based technologies have been developed, including next-generation sequencing (NGS) of a broad panel of mutations, evaluation of copy number changes, and amplification patterns. Genotyping of ctDNA by NGS is already an integral component of routine in clinical practice for a variety of tumor types. In the setting of certain advanced malignancies, such as NSCLC, ctDNA is predominantly used as a complement to tissue genotyping for the selection of biomarker-directed, first-line therapy. Nevertheless, ctDNA evaluation in patients receiving immune checkpoint inhibitors has shown both predictive and prognostic value [10,11]. Concordance of plasma TMB and tissue TMB has already been established, and aside from serving as a non-invasive biomarker when tissue is lacking, plasma TMB may have other advantages, such as comprehensively capturing tumor genomic heterogeneity, including assessment of both the primary and metastatic sites. In the clinic, ctDNA-based genotyping is also being used as a real-time tool for monitoring emergent resistance mutations in patients receiving targeted therapy [12] with direct clinical impact and the ability to alter therapy and make treatment decisions based on evolving tumor biology. In addition to these applications, the ability to repeat ctDNA assessment in a minimally invasive fashion offers a unique opportunity to use early on-treatment changes in ctDNA for real-time assessment of therapeutic response and outcome.

Another key application of ctDNA as a biomarker is in the association of molecular response assessment using plasma-based changes in ctDNA levels. The ctDNA clearance has been demonstrated as a predictor of immunotherapy response, and conversely, lack of ctDNA decrease has been associated with a higher risk of progression [13].

Here, we present the preliminary data deriving from the longitudinal analysis of ctDNA before and after treatment in a case study of patients with advanced NSCLC enrolled in the CORELAB (New prediCtive biOmaRkers of activity and Efficacy of immune checkpoint inhibitors in advanced non-small cell Lung cArcinoma) project, referring in particular to ctDNA quantification and mutational analysis by targeted NGS. CORELAB is a 4-years multicenter project funded by the Tuscany Region (Bando Ricerca Salute 2018–C44I18003190002) aimed at investigating the potential of new circulating (ctDNA) and tissue biomarkers (TMB and vessel normalization (VN)) as predictive factors of activity and efficacy of checkpoint inhibitors in patients with advanced NSCLC.

Blood samples were collected for each patient before immunotherapy and after 2, 4, 6, and 12 months or until disease progression and the mutational status of patients was evaluated by a dedicated NGS-targeted panel to investigate changes in ctDNA levels as a proxy of early tumor response to ICI for OS in a cohort of 50 patients.

## 2. Results

### 2.1. Clinicopathological Characteristics of Patients

The median age of the cohort of NSCLC patients was 70 years (range 48–83 years); 66% of the patients were males and 34% females. Thirty-eight (76%) patients were current or former smokers, while 12 (24%) had never smoked. Forty-one lung cancers (82%) were adenocarcinoma, five (10%) squamous cell carcinoma and four (8%) other histological types. All patients received pembrolizumab as an immunotherapy treatment. Thirty-two percent and 22% of the patients were respectively exposed to previous surgery or chemotherapy; in particular, five patients were subjected to both. The baseline characteristics of the patients are summarized in Table 1.

### 2.2. Total Circulating Cell-Free DNA (cfDNA)

All plasma samples were successfully processed for cfDNA extraction. The median cfDNA level at baseline (T0) was 13.62 ng/mL (range: 3.75–208.50). In 40 patients, the cfDNA level at the first follow-up point (T1) was also assessed; after 2 months of treatment, the median cfDNA level resulted in 10.93 ng/mL (range: 4.29–56.70).

Comparing cfDNA levels at baseline with those measured after two months, 4/40 (10%) patients had a reduction of at least 50%, 13/40 (32.5%) had an increase of at least 50%, while 23/40 (57.5%) had no change (reduction or increase of less than 50%).

Total cfDNA amount showed no correlation with clinical outcome.

### 2.3. Circulating Tumor DNA and Detected Variants

Cell-free DNA from 50 baseline and 40 follow-up blood samples from NSCLC patients were successfully sequenced by targeted NGS using a dedicated panel to detect lung tumor-derived cfDNA.

The mutational status of the patients under baseline conditions (T0 samples) is as follows: 19/50 (38%) patients had no variants detected in the 11 genes included in the panel, while 31/50 (62%) were mutated; in particular, 22/50 (44%) had only one mutation, 6/50 (12%) carried two mutations and 3/50 (6%) more than two mutations. The two most frequently mutated genes were *TP53* and *KRAS*, with a total of 17 and 14 mutations, respectively. *TP53* and *KRAS* were mutated in 11/50 (22%) and 9/50 patients (18%), respectively, and 4/50 (8%) patients harbor mutations in both genes at baseline. Less frequent mutations were found in other genes included in the panel: *BRAF*, *EGFR*, *MAP2K1*, *MET*, *NRAS*, and *PIK3CA* (Table 2).

The first follow-up molecular characterization was feasible for 40 out of 50 patients: 26/40 (65%) had no variants detected in any of the genes included in the panel after two months of treatment, while 14/40 (35%) were positive for mutation detection. 6/40 (15%) patients carried only one variant, 4/40 (10%) two variants, and 4/40 (10%) more than two variants. 3/40 (7.5%) patients were mutated in the *KRAS* gene, 3/40 (7.5%) were mutated in *TP53*, and 3/40 (7.5%) were mutated in both genes at T1. Other mutations were found in *BRAF*, *EGFR*, *MAP2K1*, *MET*, *NRAS*, and *PIK3CA* (Table 2). The presence of specific metastatic sites, such as brain or liver, did not correlate with the baseline ctDNA values (*p* = 0.793 and *p* = 0.447, respectively); on the contrary, patients with >2 metastatic sites had significantly higher ctDNA baseline values, reflecting the different volume tumor burden (*p* = 0.004).

### 2.4. ctDNA at Baseline (T0) and After Two Months of Immunotherapy (T1)

Sixteen out of 40 (40%) patients had no detected variants at baseline and after two months of immunotherapy; 14/40 (35%) showed a reduction in ctDNA level of at least 50% from T0 to T1 with 10 T1 samples resulting below the detection limit of our method.

**Figure 1:**
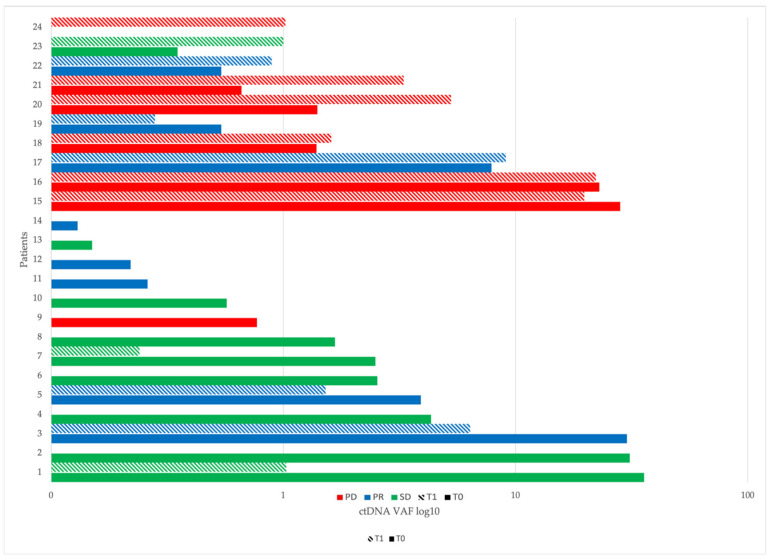
ctDNA and radiological variation between T0 (untextured) and T1(textured); patients without detectable variants at both times are not represented. Patients 1–14 had a reduction in ctDNA, patients 15–19 had stable ctDNA, and patients 20–24 had an increase in ctDNA. Bars are represented in green for patients with radiological Stable Disease (SD) per RECIST, blue for Partial Response (PR), and red for Progressive Disease (PD).

Five out of 40 (12.5%) had an increase in ctDNA level of at least 50% (one of them was wild type at baseline), and in 5/40 (12.5%) patients, the ctDNA level remained stable between the two time points (changes lower than 50%) (Figure 1).

### 2.5. ctDNA Correlation with Clinical Outcome and Survival

**Figure 2:**
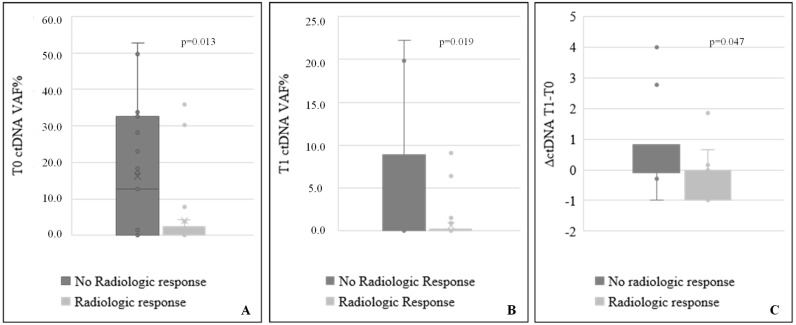
Distribution of ctDNA values at baseline and after 2 months of therapy based on radiological response. Distribution of ctDNA levels at T0 (**A**), after two months of immunotherapy, T1 (**B**) and the variation of ctDNA amount between the two time points ∆ctDNA (ctDNAT1 − ctDNAT0)/ctDNAT0; (**C**), according to the radiologic response; (Mann–Whitney test).

Statistical analysis combining ctDNA values collected at both time points with clinical outcome showed that patients without radiological response (NRR, 38%, *n* = 19) had higher ctDNA levels in plasma than patients with a positive radiological response (RR, *n* = 31). This result was confirmed regardless of the variants detected at both time points T0 (Figure 2, panel A, NRR *n* = 19, RR *n* = 31) and T1 (Figure 2, panel B, NRR *n* = 10, RR *n* = 30, *p*-values 0.013 and 0.019 respectively). Only patients without detectable ctDNA at both T0 and T1 showed a complete radiological response.

Furthermore, the delta value of ctDNA between T1 and T0 (∆ctDNA = (ctDNAT1 − ctDNAT0)/ctDNAT0) was correlated with the clinical outcome in terms of radiological response (Figure 2, panel C; NRR *n* = 10, RR *n* = 30, *p*-value 0.047).

Wild-type patients (patients without detectable variants) appeared to have better survival than those with at least one allelic variant detected, although the OS analysis did not reach full statistical significance (*p* = 0.06).

**Figure 3:**
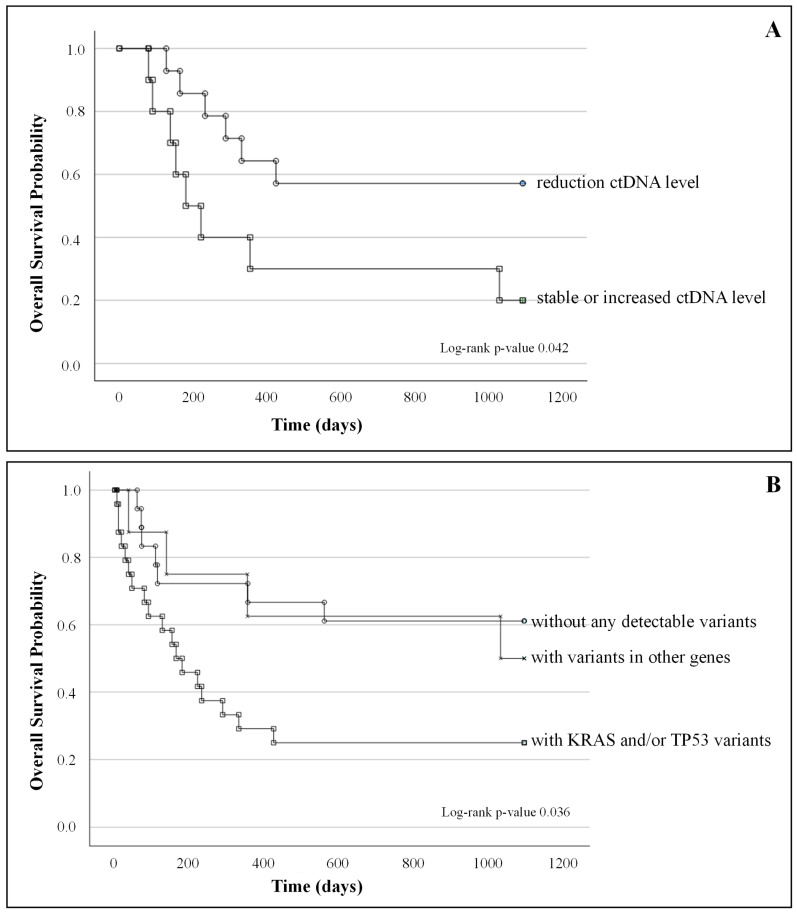
Association of molecular response with survival outcomes (**A**); association of the pre-treatment genomic status with survival outcomes (**B**).

Concerning the association between molecular response and patient survival, a log-rank test analysis performed by stratifying mutated patients into two groups according to T0-T1 ctDNA dynamics (i.e., patients with reduced ctDNA versus patients with stable or increased ctDNA) showed that OS and PFS were significantly higher in patients with ≥50% reduction in ctDNA between T0 and T1 (*p*-values 0.042 and 0.004 respectively) (Figure 3A and Supplementary Figure S1A.

At a baseline, patients with *KRAS* and/or *TP53* mutations (*n* = 24) showed a worse outcome in terms of OS than both patients carrying mutations in other genes (*n* = 8) and patients without detectable variants (*n* = 18) (*p*-value 0.036), when considered independently of PD-L1 expression (Figure 3B). These results were not confirmed with respect to PFS (*p* = 0.383, Supplementary Figure S1B. The prognostic value of *KRAS* and *TP53* mutations is strengthened by the fact that they did not result to be associated with a higher baseline incidence of brain/liver metastases or a higher number of metastatic sites in our population (*p*-values for *KRAS* = 0.158, =0.100, =0.584 and for TP53 = 0.664, 0.103, 0.666, respectively).

PD-L1 expression levels were not associated with the clinical outcome of patients (OS *p* = 0.24); interestingly, patients with *KRAS* and/or *TP53* mutations and PD-L1 expression > 50% (22% of all patients) showed a good outcome (OS), significantly different from those with PD-L1 expression below 50% (log-rank test, *p* < 0.001, Supplementary Figure S2).

(A) Kaplan–Meier survival curves of patients with a reduction in ctDNA level between T0 and T1 (*n* = 14) and patients with stable or increased ctDNA level (*n* = 10); log-rank test *p*-value = 0.42;
(B) Kaplan–Meier survival curves of patients without any detectable variants (*n* = 18), patients with *KRAS* and/or *TP53* (*n* = 24) variants, and patients with variants in other genes (*n* = 8); log-rank test *p*-value 0.036.

### 2.6. Longitudinal Monitoring by Liquid Biopsy

**Figure 4:**
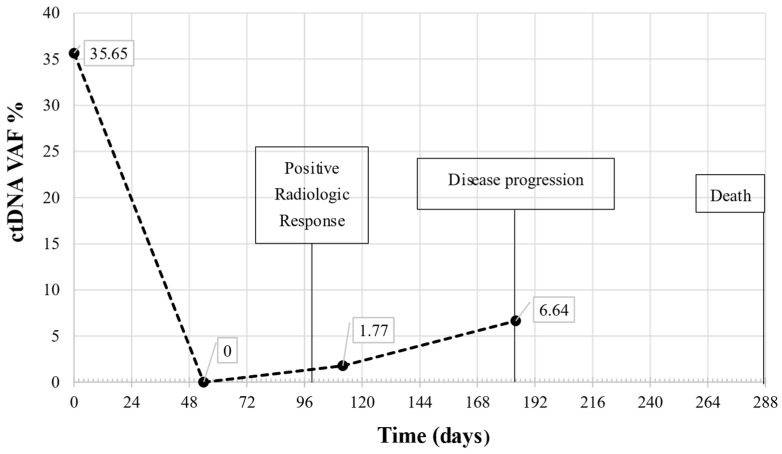
Longitudinal monitoring of an NSCLC patient with a *TP53* mutation. ctDNA longitudinal monitoring of a case study patient at T0 (day 0), T1 (day 54), T2 (day 112) and at TP (day 184). ctDNA VAF% (corresponding to a *TP53* variant) at different follow-ups are shown with associated clinical data.

The 10-month longitudinal monitoring in one patient of our study cohort is reported in Figure 4. The patient was selected as an example case monitored by ctDNA evaluation, from baseline to disease progression, with a follow-up every two months. NGS mutational analysis revealed a single variant in the *TP53* gene (*TP53* p.R273P), with a VAF% of 35.65% at baseline. The mutation was undetectable at T1, whereas a quantitatively limited new increase was observed at T2 (VAF% 1.77). Furthermore, at TP, 184 days after the start of immunotherapy, a further increase in VAF% reached 6.64%.

Regarding the association with clinical data, the patient had a radiological response (stable disease) after 100 days of immunotherapy, anticipated by the molecular response represented by undetectable ctDNA levels at T1. The patient died at 288 days from T0, after disease progression was also confirmed at the molecular level by a marked increase in ctDNA (Figure 4).

## 3. Discussion

ctDNA analysis is emerging as a new non-invasive tool to assess disease monitoring and therapeutic response, although in the context of immunotherapy, its specific application is still to be clarified [14].

In this study, we evaluated the association of plasma ctDNA presence and its mutational profile at baseline, as well as the changes emerging after 8 weeks from the beginning of therapy. The plasma mutational status of the patients was assessed by means of a targeted NGS strategy that allowed the identification of SNVs and small INDELs; the results provided a correlation with radiological response and outcomes in 50 patients with NSCLC receiving ICI.

This study was implemented in a subset of patients enrolled in the CORELAB project, a four-year project funded by the Tuscany Region, aimed at investigating the possible use of new circulating and tissue markers, such as ctDNA, TMB and vessel normalization (VN), in relation to a reference biomarker such as PD-L1 expression. In particular, we focused on the evaluation of ctDNA mutational analysis as a predictor of the activity and efficacy of checkpoint inhibitors in patients with advanced NSCLC. To date, 106 patients have undergone blood sampling at baseline and at subsequent cycles of therapy every 2 months until tumor progression.

For this purpose, we paid particular attention to the pre-analytical phase of the workflow, adopting procedures compliant with the international standard ISO 20186-3 (Molecular in vitro diagnostic examinations—Specifications for pre-examination processes for venous whole blood—Part 2: Isolated cell-free DNA) and using certified blood collection tubes containing a specific stabilizer for cfDNA analysis, having previously tested its suitability for downstream application [15]. Furthermore, we defined storage and transport conditions and adopted a standardized protocol for automated cfDNA isolation, as well as procedures for evaluating the quantity and quality of isolated cfDNA. Indeed, the lack of standardized protocols is one of the hurdles hampering the application of cfDNA analysis in routine clinical laboratories; as such, improving the pre-analytical steps to recover high-quality cfDNA is strongly needed [16].

All plasma samples were successfully processed for cfDNA extraction. No statistical association has been found between total cfDNA quantity and clinical outcomes, with most of the samples showing no variation over time within the first 2 months of therapy. In fact, although cfDNA has been reported to be generally higher in cancer patients than in healthy subjects, its use as a predictive or prognostic biomarker is still controversial [17,18]. Peng [19] reported that patients with advanced NSCLC with lower cfDNA and neutrophil/lymphocyte ratio (NLR) before treatment had a better prognosis; in contrast, increased cfDNA concentration before the third treatment, including immunotherapy, was an independent factor of disease progression in advanced NSCLC patients. These results may be useful in identifying high-risk patients and guiding treatment strategies. However, in our study, probably due to the limited number of patients, we found no evidence of a predictive or prognostic role of total cfDNA concentration in plasma. Apart from the number of patients, another reason that may justify these results is related to the type of method used for cfDNA quantification; indeed, despite the growing importance of cfDNA in oncology, there is no general consensus on protocols for cfDNA analysis, and standardization of pre-analytical and analytical procedures is necessary to obtain accurate and reproducible results in different laboratories.

For ctDNA, we estimated the amount in each sample as the sum of all VAFs for each observed tumor-related somatic mutation using a panel of 11 target genes specific to lung cancer genomic profiling. Analysis of the obtained ctDNA amount allowed the identification of patients with a molecular response (measured as a VAF decrease ≥50% between T0 and T1); we found that 35% of patients had a reduction in ctDNA levels of at least 50%, and that this molecular response was significantly associated with OS (*p* = 0.042) and to response to treatment (radiological response, *p* = 0.047). These data are in agreement with those already reported in other studies. In fact, in a retrospective study on 86 NSCLC patients treated with PD-1 inhibitors as a second or subsequent line of therapy, Guilbert et al. [20] reported that changes in ctDNA assessed by plasma-targeted sequencing can predict response to therapy; similarly, other authors, examining ctDNA changes specifically in patients who received first-line pembrolizumab-based therapies using NGS found that early ctDNA changes precede radiographic response and correlate with clinical outcomes to immunotherapy [21].

The radiological response was also statistically associated with ctDNA quantity both at the baseline and after 2 months of treatment, showing that, even at a baseline, ctDNA correlates to the clinical response, with patients with lower ctDNA levels having a better overall response rate. This potential predictive aspect of ctDNA at baseline needs to be verified in a larger cohort of patients, but it could lead to an early and useful indication in the clinic before the start of immunotherapy. Furthermore, the ctDNA delta value between 2 months after therapy and baseline was correlated as well with the clinical outcome in terms of radiologic response. These preliminary data seem to confirm that, independently from the detected variant(s) and from the absolute amount of ctDNA, its reduction is associated with a better outcome.

We detected ctDNA allelic variants at baseline in 62% of patients; patients with no detectable variants seemed to have a better outcome, but OS analysis revealed no statistically significant differences between the two groups. We can address the lack of statistical significance to the limited amount of genes included in our NGS test since Thompson et al. [22] reported a higher percentage of detected variants (93%) in ctDNA of NSCLC patients using a wider, non-lung cancer-specific panel and this can be the reason of the different percentage of total identified variants.

Among the eleven genes included in our NGS evaluation, *TP53* and *KRAS* were the most frequently mutated, while fewer common variants were found in *BRAF*, *EGFR*, *MAP2K1*, *MET*, *NRAS*, and *PIK3CA*. The detected variants reflect the mutational landscape typical of advanced NSCLC, as reported in the COSMIC (Catalogue Of Somatic Mutations In Cancer) database.

Patients with *KRAS* and/or *TP53* mutations showed the worst outcome when compared to patients without detectable variants or with mutations in other genes (log-rank test analysis, *p* = 0.036). The predictive or prognostic role of *KRAS* and *TP53* in NSCLC treated with ICI is still debated. Pavan and colleagues [23] found that patients carrying TP53 alterations had a significantly shorter OS compared to patients wild-type for TP53 and that co-mutations of TP53/STK11 or KRAS/STK11 or TP53/KRAS/STK11 had a negative impact on the OS. Even in their series, similarly to ours, such impact on PFS to immunotherapy was not confirmed in all sub-analysis. On the other hand, other groups observed an opposite trend; it was reported that *KRAS* and *TP53* mutations could serve as a positive predictive factor in guiding immunotherapy in lung adenocarcinoma, boosting PD-L1 expression and increasing tumor immunogenicity [24].

Interestingly, the OS analysis considering patients with *KRAS* and/or *TP53* mutations and PD-L1 expression > 50% (a limited number of patients representing only 22% of our cases) showed a good outcome, significantly different from those with the same mutations in plasma but PD-L1 expression less than 50% (log-rank test *p* < 0.001).

A quite similar observation was made in a selected lung cancer population composed only of high PD-L1 (≥50%) expression patients, where a positive prognostic value was suggested for *KRAS* and *TP53* co-mutations [25].

The significance of *KRAS*/*TP53* variants is further supported by the fact that, in our study, no statistically significant differences were found when considering all allelic variants with respect to PD-L1 levels. Taken together, even though our results on this topic could be affected by the limited numbers and should be considered mainly hypothesis-generating, they confirm the aggressiveness of TP53 and KRAS mutated NSCLC, while data on their predictive value to immunotherapy remains elusive.

We performed ctDNA mutational analysis over time (at baseline, after 2 and 4 months, and at disease progression) on a subgroup of patients and reported in this article the results of one patient as an example of the possible application of liquid biopsy in patient monitoring, even in the long term. In this longitudinal study, a reduction in ctDNA level between the start of immunotherapy and 2-month follow-up anticipated the radiological response; furthermore, a strong increase in the amount of ctDNA was observed at the time of disease progression, demonstrating that ctDNA is a dynamic biomarker that allows for real-time molecular monitoring of therapy efficacy in support of other medical tests such as radiological evaluation. Of note, this *TP53* mutated patient showed no detectable levels of PD-L1 in the tumor tissue and, in agreement with the previous findings, had a short survival interval from the start of immunotherapy.

## 4. Materials and Methods

### 4.1. Patients and Blood Collection

In the CORELAB study, patients with advanced (stage III/IV) NSCLC who were candidates for and treated with immune checkpoint inhibitors treatment (I/II-line treatment), according to clinical practice, were enrolled. The study was approved by the Local Ethical Committee, and written informed consent was obtained from all patients. Blood samples were collected before ICI (Time 0, T0) and when feasible, after 2, 4, 6, and 12 months of treatment (Time 1, T1; Time 2, T2; Time 3, T3; and Time 4, T4), or at the time of disease progression (TP). The cohort of patients analyzed in the present study refers to the first 50 patients enrolled in the CORELAB project.

Peripheral blood was collected in 10 mL cell-free DNA BCT (STRECK Corporate, La Vista, NE, USA) tubes.

The responders were classified as Stable Disease (SD, *n* = 13), Partial Response (PR, *n* = 14), and Complete Response (CR, *n* = 4), according to the RECIST guidelines, while the remaining patients were classified as Progressive Disease (PD, *n* = 19, including 7 clinical progressions happening before the scheduled follow-up imaging).

### 4.2. Plasma Separation, Cell-Free DNA Extraction and Quantification

Separation of plasma from whole blood was performed, according to the manufacturer’s instructions, with two centrifugation steps (10 min at 1600× *g* and 10 min at 16,000× *g*, room temperature, respectively). Plasma aliquots were stored at −80 °C until processing. DNA was extracted from 2 mL of plasma using the QIAsymphony Circulating DNA Kit (Qiagen, Hilden, Germany) on the QIAsymphony automated platform. DNA quantification was obtained using the Qubit 3.0 Fluorometer with Qubit dsDNA HS Assay Kit (Thermofisher Scientific, Waltham, MA, USA).

### 4.3. Cell-Free DNA Analysis

Samples were analyzed using the Oncomine™ Lung cfDNA Assay (Thermofisher Scientific), a dedicated panel for the detection of lung tumor-derived ctDNA single nucleotide variants (SNVs) and short indels (INDELS). In particular, more than 150 hotspots in 11 genes (*ALK*, *BRAF*, *EGFR*, *ERBB2*, *KRAS*, *MAP2K1*, *MET*, *NRAS*, *PIK3CA*, *ROS1* and *TP53)* are covered. Variant annotations, including the classification, are reported in Supplementary Table S1. The method involves the use of tag sequencing technology. Through the use of tag sequencing technology, a limit of detection (LOD) for each sample and each hotspot is calculated. In particular, the Oncomine Lung cfDNA Assay has a flexible detection limit down to a Variant Allele Frequency (VAF) of 0.1% using 20 ng of input cell-free DNA (cfDNA). The libraries were prepared according to the manufacturer’s instructions and were quantified by the Ion Library Quantitation Kit (Thermofisher Scientific) using the 7900HT system (Applied Biosystems). Template preparation for sequencing was performed using the Ion 540™ Kit-OT2 (Thermofisher Scientific) by the Ion OneTouch™ 2 System and Ion One Touch ES. Sequencing was performed on the Ion S5 using Ion 540 Chips (Thermofisher Scientific) by analyzing 24 samples per chip. Data analysis was carried out using the Ion Reporter Software (v 5.18.2.0) and the workflow Oncomine Lung Liquid Biopsy–w1.8–DNA–Single Sample.

### 4.4. ctDNA Assessment from Next Generation Sequencing Data and Molecular Response Measurement

VAF value of a detected variant was used to assess ctDNA content in each sample; when multiple variants were found (16/90 samples, 17.7%), we calculated ctDNA content as the sum of all the VAFs values for each identified variant. In most studies, both the single variant with the highest VAF [21] and the average of all variants [22] detected were used. The adoption of the sum of all the allelic variants, already reported by other authors [26,27], was made after a preliminary evaluation where VAF’s sum was compared to the highest VAF per sample and to ctDNA quantity calculated in ng/mL and no significant differences were observed.

The correlation of ctDNA dynamic changes with clinical response has been so far heterogeneous, with no general consent for what should be considered a positive or a negative response. As already reported in other immunotherapy-based studies [14,22], we defined a positive molecular response as a ≥50% reduction of ctDNA content from T0 to T1.

Data analysis parameters (depth, coverage, mapped reads) are included in Supplemental Material (Supplementary Table S1).

### 4.5. Statistical Analysis

Demographic and clinical data were analyzed using descriptive statistics. Statistical comparisons for continuous variables were performed using the Mann–Whitney test. OS, defined as the period between the date of start of ICI to the date of death, and PFS, defined as the period between the date of start of ICI and radiological/clinical progression used in Kaplan–Meier survival curves in combination with the log-rank test. Analyses were performed using the IBM SPSS Statistics software package, version number: 28.0.1.0 (142).

## 5. Conclusions

In conclusion, the data presented shows that ctDNA may be a dynamic and reliable tool for predicting and monitoring response to ICIs in patients with stage III/IV NSCLC in addition to PD-L1 expression levels with respect to both quantitative and qualitative data. Moreover, baseline ctDNA levels may help to select patients who can benefit from treatment escalation strategies or who need treatment improvement. The potential predictive role of *KRAS* and *TP53* mutations, also in association with PD-L1, may be an important aspect in the management of NSCLC patients undergoing ICI therapy. Such results need to be confirmed and verified in a larger case series.

## Figures and Tables

**Table 1:** Clinical data of the NSCLC patients included in the study.

Total number of patients	50 (100%)
Sex	
Male	33 (66%)
Female	17 (34%)
Age	(years)
Mean (SD)	69 (7.86)
Median (range)	70 (48–83)
Smoking history	
Current or former	38 (76%)
Never	12 (24%)
Histology	
Adenocarcinoma	41 (82%)
Squamous cell carcinoma	5 (10%)
Other	4 (8%)
Clinical stage	
III	6 (12%)
IV	44 (88%)
Number of metastatic sites	
1	11 (22%)
2	23 (46%)
≥3	16 (32%)
Metastatic location	
Contralateral lung	19
Pleura	4
Adrenal gland	10
Lymph nodes	29
Bones	21
Brain	13
Liver	11
Other	7
Tissue PD-L1%	
<1%	20 (40%)
1–49%	11 (22%)
≥50%	11 (22%)
Unknown	8 (16%)
Systemic Treatment	
Chemo-immunotherapy	39 (78%)
Mono-immunotherapy	11 (22%)
Immunotherapy	
Pembrolizumab	50 (100%)

Sex, Age, Smoking history, Histology, Clinical stage, Number of metastatic sites and metastatic location and Tissue PD-L1% assessed by IHC.

**Table 2:** Mutational analysis and variants annotation (from ClinVar—https://www.ncbi.nlm.nih.gov/clinvar, accessed on 10 October 2024).

Gene (NM)	CDS and AA Mutation	Cosmic ID	Type	T0 (*n*)	T1 (*n*)	Classification
*BRAF* NM_004333.6	c.1799T>A p.(Val600Glu)	COSM476	missense	2	3	pathogenic/oncogenic
*EGFR* NM_005228.5	c.2235_2249del p.(Glu746_Ala750del)	COSM6223	del	1	0	pathogenic/oncogenic
	c.2240_2257del p.(Leu747_Pro753delinsSer)	COSM12370	del	1	0	likely pathogenic
	c.2155G>A p.(Gly719Ser)	COSM6252	missense	2	2	likely pathogenic
*KRAS* NM_004985.5	c.34G>T p.(Gly12Cys)	COSM516	missense	7	5	likely pathogenic/pathogenic
	c.35G>T p.(Gly12Val)	COSM520	missense	3	0	pathogenic/oncogenic
	c.35G>C p.(Gly12Ala)	COSM522	missense	3	0	pathogenic/oncogenic
	c.37G>T p.(Gly13Cys)	COSM527	missense	1	1	likely pathogenic/oncogenic
*MAP2K1* NM_002755.4	c.167A>C p.(Gln56Pro)	COSM1235481	missense	1	2	pathogenic
	c.607G>A p.(Glu203Lys)	COSM232755	missense	1	0	likely pathogenic
	c.371C>T p.(Pro124Leu)	COSM1315861	missense	1	0	likely pathogenic/pathogenic
	c.158T>G p.(Phe53Cys)	COSM1562837	missense	0	2	na
*MET* NM_000245.4	c.3028+1G>A (p. unknown)	COSM6108461	substitution intronic	1	0	pathogenic
	c.3802A>G p.(Met1268Val	na	na	0	1	na
*NRAS* NM_002524.5	c.38G>A p.(Gly13Asp)	COSM573	missense	1	1	likely pathogenic/pathogenic
*PIK3CA* NM_006218.4	c.1624G>A p.(Glu542Lys)	COSM760	missense	2	0	pathogenic
	c.3140A>G p.(His1047Arg)	COSM775	missense	1	3	pathogenic
	c.1633G>A p.(Glu545Lys)	COSM763	missense	1	2	likely pathogenic/pathogenic
*3TP53* NM_000546.6	c.818G>C p.(Arg273Pro)	COSM43896	missense	1	0	likely pathogenic/pathogenic
	c.818G>T p.(Arg273Leu)	COSM10779	missense	1	0	pathogenic
	c.817C>T p.(Arg273Cys)	COSM10659	missense	1	0	likely pathogenic/pathogenic
	c.818G>A p.(Arg273His)	COSM10660	missense	0	2	pathogenic
	c.659A>G p.(Tyr220Cys)	COSM10758	missense	3	1	pathogenic/oncogenic
	c.742C>T p.(Arg248Trp)	COSM10656	missense	4	1	pathogenic/oncogenic
	c.743G>A p.(Arg248Gln)	COSM10662	missense	1	1	pathogenic/oncogenic
	c.733G>T p.Gly245Cys)	COSM11081	missense	1	0	pathogenic/oncogenic
	c.734G>T p.(Gly245Val)	COSM11196	missense	1	1	pathogenic/oncogenic
	c.524G>A p.(Arg175His)	COSM10648	missense	1	0	pathogenic/oncogenic
	c.711G>A p.(Met237Ile)	COSM10834	missense	1	0	pathogenic/oncogenic
	c.473G>T p.(Arg158Leu)	COSM10714	missense	1	1	likely pathogenic/pathogenic
	c.517G>T p.(Val173Leu)	COSM43559	missense	1	0	pathogenic/oncogenic

Number of mutated T0 and T1 samples for each detected variant grouped by gene and relative COSMIC ID.

## Data Availability

The raw data supporting the conclusions of this article will be made available by the authors on request.

## Supplementary Materials

The following supporting information can be downloaded at https://www.mdpi.com/article/10.3390/ijms26020611/s1.
