# Supplementary material for: Circulating Tumor Cell-Free DNA as Prognostic Biomarker in Non-Small Cell Lung Cancer Patients Undergoing Immunotherapy: The CORELAB Experience
Source: Int J Mol Sci. 2025 Jan 13;26(2):611. doi: 10.3390/ijms26020611 (PMC11766022; doi:10.3390/ijms26020611)
Supplement: Supplementary file 1 [file ijms-26-00611-s001.zip › ijms-3208916-supplementary.pdf]

Supplementary Table S1: Sequencing parameters for To and T1 samples

ID sample	Time (T0/T1)	cfDNA input for library (ng)	*Mapped Reads*	On Target (%)	Mean Depth (reads)	Median functional molecules	Molecular based uniformity (%)	Median average molecular size	R2F conversion ratio (%)
1	0	8,16	*2408989*	96,78	51125	2275	71,43	26,7	94,73
1	1	17,28	*3992227*	97,25	105884	3005	94,29	33,3	94,86
2	0	19,08	*1467825*	97,19	36050	3113	88,57	10,6	94,34
3	0	3,53	*5817650*	96,52	145140	911	91,43	146,2	97,07
3	1	2,78	*1817557*	80,25	35011	445	94,29	72,2	95,16
4	0	10,79	*8690704*	97,63	241465	2178	94,29	100,2	96,62
4	1	3,18	*2835273*	97,24	70441	1633	97,14	40	95,98
5	0	3,38	*785956*	97,16	933.8	40	45,71	8,7	72,71
5	1	2,05	*2755402*	95,75	70052	807	97,14	74,6	96,57
6	0	4,40	*3633589*	96,74	88143	899	97,14	92,7	96,48
6	1	4,21	*1822936*	86,86	35196	503	97,14	63,2	95,5
7	0	4,48	*3619012*	99,02	111930	2003	85,71	12,6	92,92
7	1	3,55	*3105195*	97,3	81345	2566	100	28,7	95,33
8	0	5,21	*1844064*	96,6	43970	996	94,29	41,5	96,21
8	1	3,65	*2637280*	95,52	54497	1140	97,14	43,6	95,53
9	0	9,32	*5074842*	96,93	128213	3081	94,29	40	95,48
10	0	3,37	*2805978*	96,54	64878	1658	94,29	37,2	95,71
10	1	6,00	*3470775*	95,69	78582	862	74,29	99,1	96,41
11	0	28,20	*6599396*	97,6	177846	15452	100	10,3	92,59
12	0	28,92	*4100771*	97,41	106714	11705	100	8,2	91,3
13	0	3,47	*598870*	96,35	13379	622	94,29	17,9	87,26
13	1	5,41	*4276088*	93,32	102189	952	97,14	98	95,86
14	0	5,00	*2341612*	96,81	58898	1455	94,29	42,5	96,22
15	0	5,78	*2910777*	91	43432	901	100	45	94,58
15	1	2,17	*2353563*	85,39	41579	415	100	92,4	96,03
16	0	2,30	*1128988*	96,12	23887	421	88,57	50,8	94,44
16	1	4,08	*3508926*	88,1	69238	691	97,14	100,5	96,41
17	0	4,40	*939185*	97,08	20880	805	91,43	26,2	92,27
17	1	2,44	*2475242*	82,87	32775	365	94,29	86	96,46
18	0	13,44	*1965245*	97,13	49986	2826	94,29	16,5	95,24
18	1	14,88	*5470456*	96,97	135047	6896	97,14	18,9	94,6
19	0	1,50	*939266*	96,83	19790	393	91,43	47,5	93,82
19	1	1,71	*1391762*	83,58	18297	246	97,14	68,9	96,01
20	0	3,95	*2580033*	81,15	31691	488	91,43	69	95,12
20	1	12,36	*1544565*	94,05	36962	1432	97,14	25,1	95,11
21	0	8,92	*2508765*	97,6	61694	3965	97,14	15,5	94,36
21	1	11,14	*5577184*	90,64	134483	1834	94,29	70,9	95,58
22	0	2,80	*2209748*	86,91	37944	540	100	64,6	95,52
22	1	5,00	*3808652*	95,18	90860	1045	94,29	88	96,42
23	0	1,99	*3283897*	96,32	74730	742	91,43	98,2	96,85
23	1	3,36	*2756647*	90,5	58722	721	100	79,1	96,31
24	0	7,61	*2408727*	91,42	52555	970	100	49,8	95,25
24	1	6,97	*3430132*	93,86	81885	1369	94,29	55,6	95,66
25	0	5,69	*2912514*	89,24	55993	1011	100	50,9	95,15
25	1	9,73	*1570436*	94,9	31542	1783	97,14	16,9	93,75
26	0	2,70	*1867597*	83,55	25488	440	97,14	54	95,83
26	1	3,35	*924829*	90,35	20168	459	100	39,5	95,5
27	0	2,39	*1723415*	95,8	34059	569	88,57	62,4	96,06
28	0	3,98	*9696899*	88,57	155724	1328	91,43	123,3	96,48
28	1	3,80	*2412860*	88,01	47240	862	100	53,9	95,75
29	0	9,04	*2950249*	96,96	78078	2357	97,14	30,6	95,58
29	1	22,68	*7692239*	94,39	203893	4436	97,14	43	95,29
30	0	2,64	*1039139*	84,29	16689	449	100	35,4	95,31
30	1	4,90	*3125546*	89,31	63098	791	97,14	77,4	96,21
31	0	1,60	*4337320*	95,51	96329	1426	100	61,8	95,56

31	1	2,50	*1915099*	90,97	37447	638	100	54,6	95,65
32	0	3,07	*2286390*	96,07	47998	1689	97,14	27	95,21
32	1	3,66	*2760513*	90,31	57514	976	100	59	95,98
33	0	7,54	*846296*	98,03	22355	1641	94,29	11	88,27
33	1	7,70	*1134398*	91,55	24602	1151	100	20,9	94,91
34	0	2,54	*3037090*	96,93	71193	818	8857,00%	86,1	96,11
34	1	7,14	*12322629*	93,65	298211	2316	97,14	126	96,48
35	0	10,31	*1128999*	97,39	29039	2177	94,29	12,5	90,6
36	0	5,93	*1759226*	90,02	37276	852	97,14	40,3	95,01
36	1	7,76	*1458251*	88,74	30770	1349	100	21	94,46
37	0	4,08	*2693374*	84,23	44815	517	100	79,1	95,72
37	1	3,76	*558672*	97,28	13140	468	94,29	25,6	94,97
38	0	3,01	*2448360*	84,62	41734	466	97,14	83,5	95,64
38	1	3,26	*986171*	87,2	14757	503	97,14	26	93,79
39	0	8,22	*3039368*	93,23	74312	1365	100	48,3	94,77
39	1	11,33	*5289407*	96,52	138292	1730	100	75,6	97,61
40	0	7,26	*4014881*	88,98	58583	2539	91,43	21,8	91,19
40	1	8,66	*2131386*	98,49	60504	1878	100	27,3	97,3
41	0	3,37	*3328936*	76,31	57975	1134	100	48	94,49
41	1	2,57	*1064697*	83,69	14651	219	100	60,4	97,13
42	0	6,34	*4589895*	79,59	89740	1514	97,14	56,2	95,75
42	1	2,92	*1211698*	85,32	18734	243	100	68,7	97,52
43	0	4,67	*4976242*	74,37	90183	1262	97,14	66,8	96,03
43	1	5,40	*2250110*	93,29	51522	895	97,14	54,4	97,46
44	0	6,12	*1743741*	86,47	39853	1269	100	28,9	95,14
44	1	4,93	*958634*	88,61	16603	784	100	19,3	93,8
45	0	11,35	*1474648*	95,99	36872	2117	100	16	93,71
45	1	3,48	*973007*	81,76	11367	291	97,14	36,6	93,9
46	0	83,40	*1119655*	96,5	28758	4208	97,14	5,1	80,34
47	0	48,48	*33740994*	95,38	858592	10793	91,43	83,8	95,61
48	0	8,38	*10541795*	87,78	207206	2187	94,29	97,4	95,83
48	1	14,04	*5252086*	93,42	125233	2,874	100	40,5	95,06
49	0	6,73	*1339299*	89,4	28476	1025	100	25,5	94,41
49	1	4,54	*2407934*	86,44	39137	775	100	47,2	95,22
50	0	8,23	*2639579*	91,7	60449	1410	100	39,9	94,62
	**MIN**	**1,50**	***558672,00***	**74,37**	**11367,00**	**2,87**	**45,71**	**5,10**	**72,71**
	**MAX**	**83,40**	***33740994,00***	**99,02**	**858592,00**	**15452,00**	**100,00**	**146,20**	**97,61**
	**Mean**	**7,86**	***3326336,16***	**91,91**	**75545,83**	**1716,43**	**95,46**	**51,18**	**94,70**
	**SD**	**10,55**	***3892642,44***	**5,70**	**99029,61**	**2339,50**	**7,23**	**30,35**	**3,29**
	**Median**	**4,91**	***2492003,50***	**93,76**	**52555,00**	**1018,00**	**97,14**	**47,35**	**95,49**

Note to Supplementary Table S1: Sequencing parameters for To and T1 samples

Median functional molecules: median coverage based on number of molecules (functional molecules)

Median average molecular size: median number of reads with a particular molecular tag

R2F conversion ratio (%): percentage of reads contributed to functional molecules

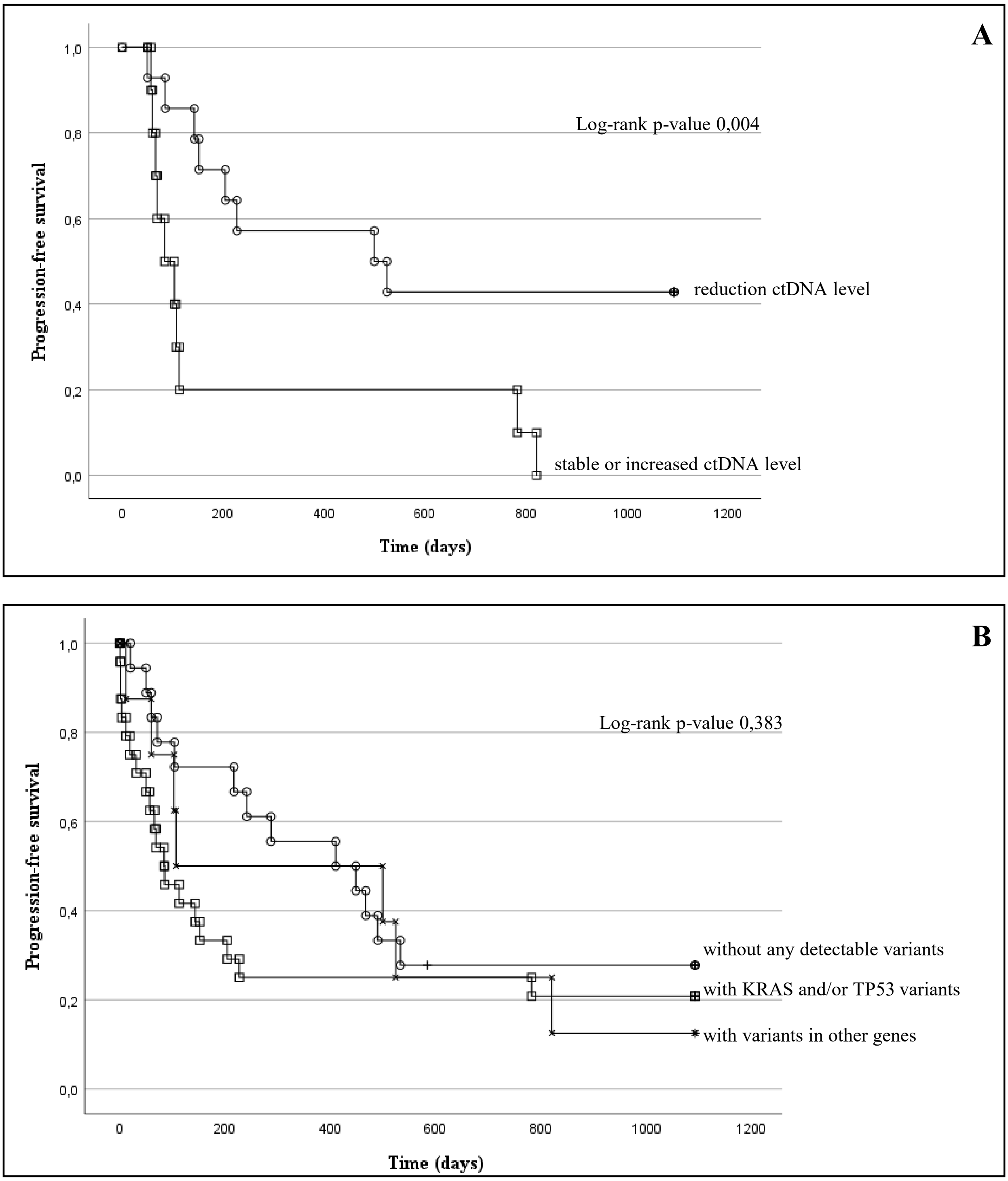

Supplementary Figure S1 Association of molecular response with PFS A); Association of the pre-treatment genomic status with PFS B).

A) Kaplan-Meier survival curves of patients with a reduction in ctDNA level between T0 and T1 (n=14) and patients with stable or increased ctDNA level (n=10); log rank test p-value = 0,004. **B**) Kaplan-Meier survival curves of patients without any detectable variants (n=18), patients with *KRAS* and/or *TP53* (n=24) variants and patients with variants in other genes (n=8)); log rank test p-value 0,383.

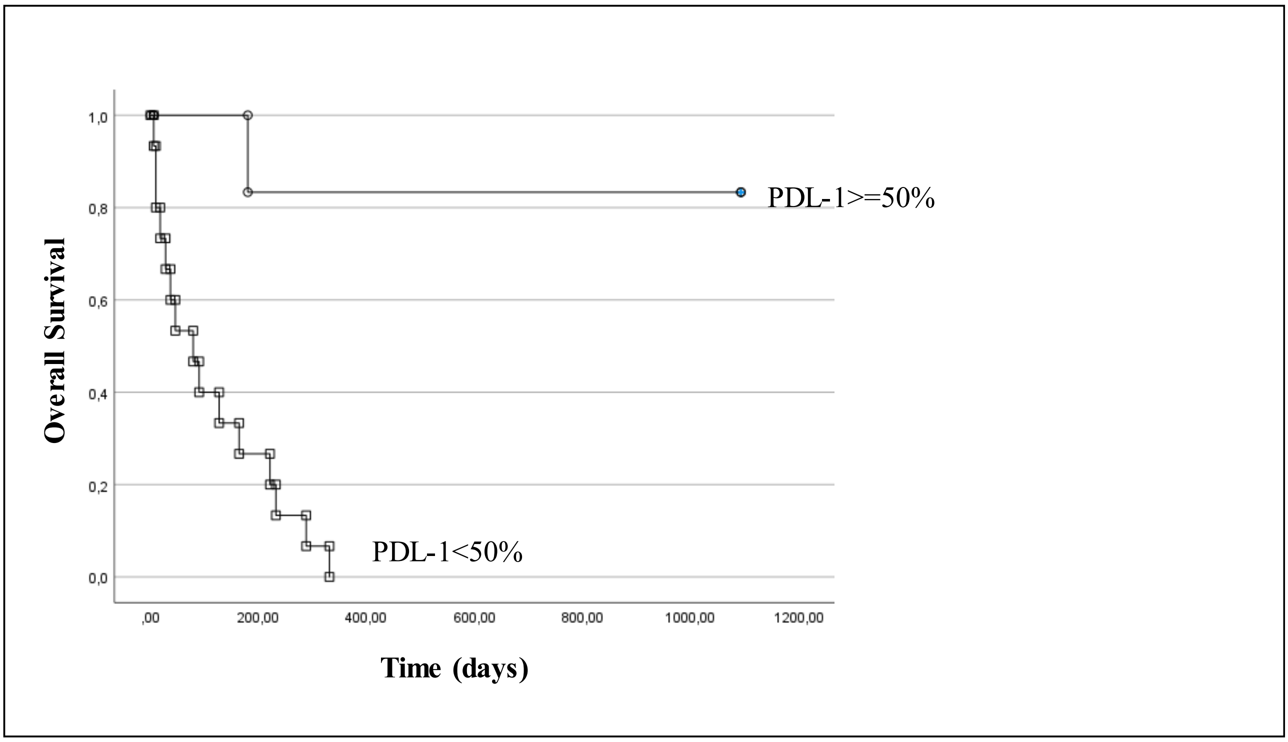

Supplementary Figure S2. Association of KRAS and/or TP53 mutated patients and PDL1 levels

Association of the pre-treatment genomic status with survival outcomes**.** Kaplan-Meier survival curves of patients with *KRAS* and/or *TP53* mutations and PDL1 expression ≥50% (n= 6) and <50% (n=15); log rank test p-value 0.001.
